# Mucus plugging on computed tomography and chronic bronchitis in chronic obstructive pulmonary disease

**DOI:** 10.1186/s12931-021-01712-0

**Published:** 2021-04-17

**Authors:** Victor Kim, Wojciech R. Dolliver, Hrudaya P. Nath, Scott A. Grumley, Nina Terry, Asmaa Ahmed, Andrew Yen, Kathleen Jacobs, Seth Kligerman, Alejandro A. Diaz, the COPDGene Investigators, James D. Crapo, Edwin K. Silverman, Barry J. Make, Elizabeth A. Regan, Terri Beaty, Ferdouse Begum, Peter J. Castaldi, Michael Cho, Dawn L. DeMeo, Adel R. Boueiz, Marilyn G. Foreman, Eitan Halper-Stromberg, Lystra P. Hayden, Craig P. Hersh, Jacqueline Hetmanski, Brian D. Hobbs, John E. Hokanson, Nan Laird, Christoph Lange, Sharon M. Lutz, Merry-Lynn McDonald, Margaret M. Parker, Dmitry Prokopenko, Dandi Qiao, Elizabeth A. Regan, Phuwanat Sakornsakolpat, Edwin K. Silverman, Emily S. Wan, Sungho Won, Juan Pablo Centeno, Jean-Paul Charbonnier, Harvey O. Coxson, Craig J. Galban, MeiLan K. Han, Eric A. Hoffman, Stephen Humphries, Francine L. Jacobson, Philip F. Judy, Ella A. Kazerooni, Alex Kluiber, David A. Lynch, Pietro Nardelli, John D. Newell Jr., Aleena Notary, Andrea Oh, Elizabeth A. Regan, James C. Ross, Raul San Jose Estepar, Joyce Schroeder, Jered Sieren, Berend C. Stoel, Juerg Tschirren, Edwin Van Beek, Bram van Ginneken, Eva van Rikxoort, Gonzalo Vegas Sanchez-Ferrero, Lucas Veitel, George R. Washko, Carla G. Wilson, Robert Jensen, Douglas Everett, Jim Crooks, Katherine Pratte, Matt Strand, Carla G. Wilson, John E. Hokanson, Gregory Kinney, Sharon M. Lutz, Kendra A. Young, Surya P. Bhatt, Jessica Bon, Alejandro A. Diaz, MeiLan K. Han, Barry Make, Susan Murray, Elizabeth Regan, Xavier Soler, Carla G. Wilson, Russell P. Bowler, Katerina Kechris, Farnoush Banaei-Kashani, Jeffrey L. Curtis, Perry G. Pernicano, Nicola Hanania, Mustafa Atik, Aladin Boriek, Kalpatha Guntupalli, Elizabeth Guy, Amit Parulekar, Dawn L. DeMeo, Alejandro A. Diaz, Lystra P. Hayden, Brian D. Hobbs, Craig Hersh, Francine L. Jacobson, George Washko, R. Graham Barr, John Austin, Belinda D’Souza, Byron Thomashow, Neil MacIntyre Jr., H. Page McAdams, Lacey Washington, Eric Flenaugh, Silanth Terpenning, Charlene McEvoy, Joseph Tashjian, Robert Wise, Robert Brown, Nadia N. Hansel, Karen Horton, Allison Lambert, Nirupama Putcha, Richard Casaburi, Alessandra Adami, Matthew Budoff, Hans Fischer, Janos Porszasz, Harry Rossiter, William Stringer, Amir Sharafkhaneh, Charlie Lan, Christine Wendt, Brian Bell, Ken M. Kunisaki, Russell Bowler, David A. Lynch, Richard Rosiello, David Pace, Gerard Criner, David Ciccolella, Francis Cordova, Chandra Dass, Gilbert D’Alonzo, Parag Desai, Michael Jacobs, Steven Kelsen, Victor Kim, A. James Mamary, Nathaniel Marchetti, Aditi Satti, Kartik Shenoy, Robert M. Steiner, Alex Swift, Irene Swift, Maria Elena Vega-Sanchez, Mark Dransfield, William Bailey, Surya P. Bhatt, Anand Iyer, Hrudaya Nath, J. Michael Wells, Douglas Conrad, Xavier Soler, Andrew Yen, Alejandro P. Comellas, Karin F. Hoth, John Newell Jr., Brad Thompson, MeiLan K. Han, Ella Kazerooni, Wassim Labaki, Craig Galban, Dharshan Vummidi, Joanne Billings, Abbie Begnaud, Tadashi Allen, Frank Sciurba, Jessica Bon, Divay Chandra, Carl Fuhrman, Joel Weissfeld, Antonio Anzueto, Sandra Adams, Diego Maselli-Caceres, Mario E. Ruiz, Harjinder Singh

**Affiliations:** 1 Lewis Katz School of Medicine at Temple University, 3401 North Broad Street, 785 Parkinson Pavilion, Philadelphia, PA 19140 USA; 2 Division of Pulmonary and Critical Care Medicine, Brigham and Women’s Hospital, Boston, MA USA; 3 Department of Radiology, University of Alabama at Birmingham, Birmingham, AL USA; 4 Department of Radiology, University of California San Diego, San Diego, CA USA

## To the Editor,

Chronic bronchitis (CB) is associated with more dyspnea, increased respiratory exacerbations, reduced exercise capacity, and mortality [1]. The hallmark of CB is mucus overproduction and goblet cell hyperplasia [2]. Mucus plugging (MP) on CT scan has been associated with decreased lung function, worse health-related quality of life, and amount of emphysema in Chronic Obstructive Pulmonary Disease (COPD) [3]. How it relates to CB in smokers with COPD is unclear. We hypothesized that those with MP are more likely to have CB compared to those without MP.

We analyzed 2089 randomly chosen subjects from the Genetic Epidemiology Study of COPD (COPDGene) [4]. Briefly, this cohort was comprised of African American and non-Hispanic White current and former smokers (≥ 10 pack year history) 45–80 years of age with COPD. Measurement of MP has been described previously [3]. A mucus plug was defined as an opacity that completely occludes the lumen, regardless of the airway size, orientation or generation. Major airways such as the trachea, main stem and lobar bronchi were excluded. In a standardized fashion, all airway paths were examined in each out of 18 bronchopulmonary segments of both lungs. An MP score was generated for each CT scan as an aggregation of the number of bronchopulmonary segments with MP (0–18). If a CT scan required more than one reading, the final MP score was an average of the scores from two or three readers.

We divided subjects into those with at least one airway plugged with mucus (MP+) and those without MP (MP−). Additionally, based on prior research in asthma showing that mucus plugging of ≥ 4 segments was associated with worse lung function [5], we divided subjects into those with an MP score ≥ 4 and compared them to those with an MP score < 4. We compared subject characteristics between groups with either an unpaired *t* test or Chi-square test. Odds ratios for CB were calculated with MP scores using multivariable logistic regression models. Covariates included demographics, lung function, smoking, and radiologic parameters.

Subject characteristics are presented in Table 1. 658 (31.5%) subjects had mucus plugging on CT scan. 133 (6.4%) subjects had an MP score of ≥ 4. Compared to the MP− group, the MP+ group was more likely to have CB by either the classic definition (cough and phlegm for at least 3 months/year for 2 consecutive years, 29.2 vs. 22.9%) or the Saint George’s Respiratory Questionnaire (SGRQ) definition (cough and phlegm for the past 4 weeks almost every day or several days a week, 39.1 vs. 25.4%). In the MP ≥ 4 group, the differences were more significant with 57.7% and 41.4% of the subjects having SGRQ CB and classic CB, respectively, compared to 27.6% and 23.8% of the MP < 4 group. In those with classic CB (n = 519), 36.9% of them had MP compared to 29.7% of those without classic CB (n = 1570, p = 0.002). 10.6% of the classic CB group had an MP score ≥ 4 compared to 5.0% of those without classic CB (p < 0.0001). Similar values were found when comparing those with and without SGRQ CB (38.8 vs. 25.1%, p < 0.0001 for MP+ vs. MP− and 11.4 vs. 3.5%, p < 0.0001 for MP ≥ 4 vs. MP < 4). Dyspnea, health-related quality of life, and lung function were worse in the MP+ group (p < 0.05 for all). Percent emphysema, percent gas trapping, airway wall thickness, exacerbation frequency and severe exacerbation frequency were all significantly higher in the MP+ group (p < 0.05 for all). Except for %emphysema, findings were similarly significant in the MP ≥ 4 group compared to the MP < 4 group.

**Table 1:** Subject characteristics

	MP+ (n = 658)	MP− (n = 1431)	p	MP ≥ 4 (n = 133)	MP < 4 (n = 1956)	p
Age (years)	64.7 ± 8.8	62.6 ± 8.5	< 0.0001	64.7 ± 8.8	63.2 ± 8.6	0.064
BMI (kg/m^2^)	27.1 ± 5.8	28.2 ± 6.0	< 0.0001	26.2 ± 6.3	28.0 ± 6.0	0.001
Gender (% male)	51.1	57.2	0.009	47.4	55.8	0.059
Race (% non-Hispanic White)	81.6	74.3	< 0.0001	85.7	76	0.010
Currently smoking (%)	36.5	42.8	0.007	45.9	40.4	0.218
Smoking history (pack-years)	52.6 ± 29.2	50.3 ± 25.7	0.071	50.8 ± 30.6	51.1 ± 26.6	0.920
Phlegm 3 months or more (%)	44.4	36.1	< 0.0001	60.5	37.2	< 0.0001
Chronic bronchitis classic def (%)	29.2	22.9	0.002	41.4	23.8	< 0.0001
Chronic bronchitis SGRQ def (%)	39.1	25.4	< 0.0001	57.7	27.6	< 0.0001
SGRQ	42.3 ± 21.6	33.3 ± 22.7	< 0.0001	45.4 ± 21.9	35.4 ± 22.7	< 0.0001
mMRC	2.25 ± 1.41	1.72 ± 1.46	< 0.0001	2.45 ± 1.33	1.85 ± 1.47	< 0.0001
FEV1% predicted	47.5 ± 20.8	61.8 ± 22.9	< 0.0001	42.8 ± 18.7	58.3 ± 23.2	< 0.0001
FVC% predicted	75.9 ± 20.0	84.8 ± 19.9	< 0.0001	73.2 ± 17.7	82.6 ± 20.3	< 0.0001
FEV1/FVC	0.46 ± 0.13	0.54 ± 0.13	< 0.0001	0.44 ± 0.13	0.53 ± 0.13	< 0.0001
%emphysema	16.0 ± 13.6	11.2 ± 11.9	< 0.0001	14.5 ± 13.0	12.6 ± 12.6	0.109
%gas trapping	44.6 ± 19.7	32.3 ± 19.8	< 0.0001	46.4 ± 18.5	35.6 ± 20.6	< 0.0001
Pi10	3.74 ± 0.14	3.68 ± 0.13	< 0.0001	3.81 ± 0.16	3.69 ± 0.13	< 0.0001
WA%, segmental	62.8 ± 3.0	62.0 ± 3.0	< 0.0001	64.0 ± 3.0	62.2 ± 3.0	< 0.0001
Exac freq in year prior (no./pt/year)	0.79 ± 1.28	0.54 ± 1.10	< 0.0001	1.11 ± 1.56	0.59 ± 1.13	< 0.0001
% With sev exac in year prior (%)	23.1	16.8	0.001	29.3	18.0	0.001

Data are expressed as mean ± SD or percent

Odds ratios (OR) for classic CB and SGRQ CB are shown in Table 2. MP+ was associated with an OR of 1.21 and 1.38 for classic CB and SGRQ CB, respectively when adjusting for demographics, lung function, smoking, and CT measures of emphysema and airway wall thickness. Similarly, MP ≥ 4 was associated with an OR of 1.67 and 2.05 for classic CB and SGRQ CB, respectively.

**Table 2:** Odds ratios (OR) for chronic bronchitis

Classic CB	Model 1	Model 2	Model 3	Model 4
MP+	1.48 (1.20, 1.83)	1.29 (1.03, 1.61)	1.20 (0.96, 1.51)	1.21 (0.95, 1.53)
MP ≥ 4	2.38 (1.65, 3.44)	2.06 (1.41, 2.99)	1.79 (1.22, 2.64)	1.67 (1.12, 2.49)
SGRQ CB				
MP+	2.07 (1.60, 2.68)	1.50 (1.14, 1.97)	1.43 (1.07, 1.90)	1.38 (1.03, 1.85)
MP ≥ 4	3.68 (2.29, 5.91)	2.50 (1.53, 4.10)	2.28 (1.35, 3.85)	2.05 (1.20, 3.49)

Data represent OR (95% CI). Covariates: Model 1: age, gender, race. Model 2: age, gender, race, and FEV1%predicted. Model 3: age, gender, race, FEV1% predicted, current smoking and pack year history. Model 4: age, gender, race, FEV1% predicted, current smoking and pack year history, Pi10 and %emphysema

These data also show that more subjects with MP, looking at two different thresholds (MP+ versus—and MP ≥ 4 versus < 4) had CB using two different definitions. Additionally, regardless of the presence or absence of CB, those with MP have worse symptoms, health-related quality of life and lung function compared to those without MP, showing that radiographic assessment of MP provides complementary information to the clinical assessment of CB.

Plugging of the small airways is a central pathologic feature of COPD. In a large study of pathologic specimens, patients with worse lung function were more likely to have small airway mucus luminal occlusion [6]. Radiologically, MP has been linked to severity of airflow obstruction, eosinophilic inflammation, and interleukin-4 blood levels in asthma [5, 7]. In two smaller studies, MP was associated with worse lung function and health-related quality of life in smokers with and without COPD, but neither CB nor eosinophilia [3, 8]. To date, this is the first study finding a significant association between mucus plugging and CB in a large cohort of COPD subjects. While the percent of our MP+ subjects who had CB was similar compared to prior studies, our findings likely differ because of the size of our cohort and differences in subject characteristics (other studies included never smokers and smokers without airflow obstruction).

The relationship between mucus plugging and emphysema is an interesting one. More mucus plugs have been found in COPD subjects with worse airflow obstruction; additionally, more emphysema is seen in COPD patients with worse airflow obstruction. Previous smaller studies in smokers with and without COPD have found weak but significant correlations between mucus plugging scores and percent emphysema [3, 8]. Our analysis was restricted to those with COPD, and we found statistically different amounts of percent emphysema between those with and without mucus plugs but the difference was not great. This suggests that mucus plugging may play a role in the pathophysiology of airflow obstruction in COPD, independent of emphysema.

We found that using a high threshold for MP scores (≥ 4) was significantly associated with CB, particularly when defined using the SGRQ. This may be related to the SGRQ CB definition identifying “active” symptoms (cough and phlegm in the past 4 weeks), compared to the classic CB definition which is better at identifying chronic symptoms (2 years). However, the higher threshold of MP was only present in 6.4%, suggesting that using any MP as a threshold may be more practical. Regardless of the definition, in this well-characterized cohort of COPD subjects, MP on CT scan was significantly associated with CB. Compared to those without MP, those with MP had worse symptoms, health-related quality of life, and lung function regardless of the presence or absence of CB. The findings suggest that imaging is a useful tool to objectively measure mucus dysfunction, a central pathophysiologic feature of CB. Further study is necessary to identify the consequences of MP on other patient reported outcomes.

## Data Availability

The datasets used and/or analysed during the current study are available from the corresponding author on reasonable request.
